# Reactive oxygen species and nitric oxide induce senescence of rudimentary leaves and the expression profiles of the related genes in *Litchi chinensis*

**DOI:** 10.1038/s41438-018-0029-y

**Published:** 2018-05-01

**Authors:** Haifang Yang, Hye-Ji Kim, Houbin Chen, Yong Lu, Xingyu Lu, Congcong Wang, Biyan Zhou

**Affiliations:** 1 College of Horticulture, South China Agricultural University, Guangzhou, 510642 China; 2 Department of Horticulture and Landscape Architecture, Purdue University, West Lafayette, IN 47907-2010 USA

## Abstract

Litchi is one of the most important subtropical evergreen fruit trees in southern Asia. Previous studies indicated that high-temperature conditions encourage growth of rudimentary leaves in panicles and suppress flowering. We have demonstrated that methyl viologen dichloride hydrate (MV) and sodium nitroprusside (SNP) promoted flowering in litchi partially by inhibiting the growth of rudimentary leaves via reactive oxygen species (ROS) and nitric oxide (NO). In the present study, we examined the microstructure and ultrastructure, programmed cell death (PCD) ratio, nuclei morphology of the rudimentary leaves, and the expression of senescence-related genes after the treatment with ROS or NO. The results showed that chromatins of the ROS- or NO-treated cells in the rudimentary leaves were condensed. Fusion of the cytoplasm-digesting vesicles with the vacuole and degradation of cytoplasm forming scattered debris were found in those of the treated cells. Treatment with ROS or NO increased the cell PCD ratio. Morphology of the nuclei stained by propidium iodide (PI) showed that nuclei shape became irregular after the ROS or NO treatment. Further, the expression levels of *LcRboh*, *LcMC-1-like*, and *LcPirin* were higher in the ROS- and NO-treated rudimentary leaves than those in the control ones, suggesting that these genes may be involved in the ROS and NO-induced senescence and abscission of the rudimentary leaves in litchi. Our results suggested that ROS and NO play an important role in inducing the senescence of the rudimentary leaves, and ROS- and NO-induced PCD may be involved in the regulation of the rudimentary leaf growth in litchi.

## Climate change: tricking the trees

Warmer winter temperatures suppress flowering of litchi trees, threatening fruit production, but researchers have found another way to trigger litchi trees to reproduce. In litchi, spring buds contain both flower and leaf primordia, and cold cues the plants to shed the young leaves, allowing the flower primordia to develop. With recent warmer winters, the leaves are crowding out the flowers. Biyan Zhou at South China Agricultural University investigated whether spraying with compounds that make the trees produce stress-signaling molecules, such as nitrous oxide, would mimic the effects of a cold winter. Following treatment, the trees shed their rudimentary leaves and flowers developed. Microscopic examination of buds showed that the leaf shedding process looked similar to cold-triggered shedding. This study offers a method to induce litchis to flower and fruit, even in a changing climate.

## Introduction

Litchi is one of the most important evergreen fruit trees in southern Asia. Low winter temperature is indispensable for litchi flower induction^1,2^. Under normal winter conditions with a prolong exposure to low temperature, the litchi apical buds break and panicle primordia emerge when air temperature and soil moisture increase in the following spring. The buds at this stage are mixed buds containing axillary or apical panicle primordia, leaf primordia, and rudimentary leaves. Under normal climate conditions, the growth of panicle primordia prevail and the rudimentary leaves abscise. However, in the context of global warming and climate change, the mixed buds may be exposed to excessively high temperature in spring, and the rudimentary leaves develop into fully expanded leaves, inhibiting panicle primordia from development. This can be a significant threat to litchi flowering^3^, and therefore, it is important to encourage panicle development by suppressing the growth of rudimentary leaves.

It has been demonstrated that environmental stresses confer an evolutionary advantage to plant species, since it hastens reproduction and ensures the survival of the species under adverse environmental conditions^4^. Stress induces reactive oxygen species (ROS) and nitric oxide (NO) accumulation in plants^5,6^. ROS and NO are the key signaling molecules involved in plant responses to both biotic and abiotic stresses^7,8^. We have previously shown that NO and ROS promoted reproductive growth in litchi by inhibiting the growth of rudimentary leaves and enhancing the expression of *LcLFY* and *LcAP1*^9,10^.

Leaf senescence is a genetically programmed cell death (PCD) process naturally occurring during the final stage of developmental process^11,12^. Besides the naturally occurring senescence, leaves may be induced to senescence before maturity in response to environmental stimuli such as drought and excessively high or low temperature conditions^13,14^. Intracellular H_2_O_2_, a main ROS component, accumulates at the onset of leaf senescence with the parallel down regulation of H_2_O_2_-scavenging enzymes. The accumulated data suggest that ROS and NO may function in elaborating cell death^15,16^. Abscission is the last stage of senescence. It can enable senescent organs, which are no longer needed to shed^17^. In our previous studies, we found that controlling the growth of the rudimentary leaves in the leafy panicles played an important role in floral development^3^. Further, we found that methyl viologen dichloride hydrate (MV), the ROS producer, and sodium nitroprusside (SNP), the NO donor, could effectively control the growth of the rudimentary leaves. We then identified many ROS and NO-responsive genes of leaves and floral buds by a suppression subtractive hybridization library screen and digital gene expression assay^18–20^. However, the mechanisms as to how ROS and NO control the growth of rudimentary leaves need further investigation.

In the present study, we investigated the microstructure and ultrastructure, cell PCD ratio, and nuclei morphology of the rudimentary leaves after ROS or NO treatment using MV and SNP, respectively. Expression of the related genes was also determined after the treatments. We aimed to provide basic materials for the rudimentary leaf control underling flowering.

## Materials and methods

### Plant material and experimental procedures

Commercially cultivated 12-year-old litchi trees cv. Nuomici grafted onto cv. Huaizhi were selected. The trees were grown at the experimental orchard of South China Agricultural University (lat. 23°9′ N, long. 113°21′ E). Three trees of similar size with new shoots were selected as replicates. The new shoots in each tree were divided into three groups. Each groups contained about 100 new shoots. The new shoots in each group were uniformly sprayed with either water as control, 3 mM SNP (Shanghai Chemical Reagent Co., Ltd, Shanghai, China), or 120 µM MV (Sigma, St Louis, MO, USA) for one time. The doses selected for the MV and SNP treatments were according to Zhou et al.^10^. Either the chemical or water was applied as new shoot spray to runoff using a pressure sprayer. ROS or NO imaging was carried out after 10 h of MV or SNP treatment as described below. Rudimentary leaves were collected at 0 d to 9 d after the treatment for microstructure and nuclei morphology observation, and for the determination of cell PCD ratio and gene expression.

For ultrastructure observation, 4-year-old air-layered trees (*Litchi chinenesis* cv. Nuomici) were grown in 30-L pots with loam, mushroom cinder, and coconut chaff (v:v:v, 3:1:1). Uniform potted trees (the average height of 1 m containing about 30 terminal shoots) were selected for the experiment. Trees were subjected to low temperature for about 8 weeks for floral induction. When panicle primordia emerged, the potted trees were transferred to a growth chamber maintained at day/night temperature of 28/23 °C with 12-h photoperiod to encourage growth of the rudimentary leaves. Nine trees were evenly divided into three groups and sprayed one time with either water, 120 μM MV, or 2 mM SNP. Either the chemical or water was applied as new shoot spray to runoff using a pressure sprayer. Sections of the stem with petiole bases of the rudimentary leaves were excised at 0, 3, 6, and 9 d after MV treatment, and at 0, 2, and 5 d after SNP treatment.

### Hydrogen peroxide and NO imaging

A small piece of tissue including stem and petiole bases were excised, dissected lengthwise according to the method of Zhou et al.^10^. Then the tissues were incubated in buffer (50 mM Tris and 50 mM KCl, pH 7.2) containing either 50 μM dichlorofluorescin diacetate (DCFH-DA, Sigma), a ROS fluorescent probe^21^, or 10 μM NO indicator dye diaminofluorescein diacetate (DAF-2DA, Sigma)^22^, and 1% (v/v) Triton X-100 for 30 min. After dye incubation, the tissues were washed with buffer (50 mM Tris, 50 mM KCl, pH 7.2) for three times. ROS or NO distribution indicated by green fluorescent signal was acquired at excitation of 488 nm and collected in 515–560 nm wavelength range using a TCS SP2 confocal microscope system (Leica Microsystems GmbH, Wetzlar, Germany).

### Light microscopy

Tissues for microstructure observation were collected according to Yang et al.^23^. Sections of the stem consisting of a petiole base of the rudimentary leaves and an axillary bud from a new shoot were excised, vacuum penetrated, and fixed with 4% polyformaldehyde for 4 h. Then, 4% ethylenediamine was added to the buffer for tissue softening. The tissues were dehydrated in a graded ethanol series from 30 to 100% (v/v), with a final change to xylene, and then embedded in paraffin. After processing, the tissues were sectioned in 6–8 μm using a microtome (Leica RM2235, Leica Instruments, Nussloch, Germany). Then, the sections were stained with hematoxylin eosin, observed under a light microscope (Leica DMLB, Leica GmbH, Bensheim, Germany).

### Electron microscopy

Tissue collection for ultrastructure observation was carried out according to Zhou et al.^3^. Sections of the stem with a petiole base of the rudimentary leaves from a new shoot were cut. The tissues were vacuum penetrated in 4% glutaraldehyde and fixed for 4 h. Then, the tissues were washed with 0.2 mM phosphate buffer (pH 7.2) for six times, and incubated in 1% (w/v) osmium tetroxide. After rinsing with phosphate buffer, the tissues were dehydrated in series of ethanol solutions from 50 to 100% (v/v) and embedded in araldite resin. Ultra-thin (80 nm) sections were longitudinally cut using a Leica Ultracut UCT ultramicrotome (Leica Mikrosysteme Gmbh, Vienna, Austria). The sections were double stained with uranyl acetate and lead citrate, mounted on copper grids and observed under a transmission electron microscope (Philips EM400).

### Determination of cell PCD ratio

Cell PCD ratio was determined according to the method of Dolezel et al.^24^. Samples of 1 g fresh rudimentary leaves were washed in ice cold water followed by the addition of 2 mL of ice cold nuclear extraction buffer, which contained 20 mM Tris-HCl (pH 7.5), 4 mM MgCl_2_, 2 mM Na_2_EDTA, 86 mM NaCl, 10 mM sodium metabisulfite, 1% PVP-10 (Sigma), and 1% (v/v) Triton X-100. The leaf tissues were then chopped up, kept in ice cold buffer for 10 min, and filtered through a 50-μm nylon mesh. The filtrates were centrifuged at 800 × *g* at 4 °C for 5 min. The supernatants were removed and the residues were re-suspended in 400 μL nuclear extraction buffer. The suspensions were filtered through 38-μm nylon mesh. The filtrates were added with RNase A and propidium iodide (PI) buffer at a final concentration of 50 mg L^−1^, and incubated at room temperature for 10 min. The suspensions were used for determination of cell PCD ration by a flow cytometer (FCM; Cell Lab Quanta™ SC Flow Cytometer, Beckman Coulter, Miami, USA). PCD ratio (%) was given by the expression 100× numbers of nuclei in the PCD cells/total numbers of nuclei scanned.

### PI labeling assay

Nuclei were prepared as described above and 10 μL of the final suspension was pipetted onto a microscope slide and covered with a coverslip. Images were visualized by fluorescence microscopy (Leica DM1000, Leica Microsystems GmbH, Wetzlar, Germany, excitation 535 nm, emission 615 nm).

### Analysis of gene expression by quantitative reverse transcriptase-PCR (qRT-PCR)

Total RNA from rudimentary leaves was isolated, and used to generate the first-strand complementary DNA (cDNA) according to the method of Liu et al.^18^. Expression analysis was performed at transcript level, which was determined by qRT-PCR using single-stranded cDNA as a template. All the primers are shown in Supplementary Table 1. The reference gene was the litchi homolog *Actin* (accession number: HQ588865.1). Quantitative PCR (qPCR) was performed on the CFX96 Optical System (Bio-Rad) using a SYBR Green based qPCR assay. The PCR cycles were run as described by Yang et al.^23^: 95 °C for 3 min, then 40 cycles of 95 °C for 15 s and 60 °C for 30 s in 96-well optical reaction plates (Bio-Rad, Hercules, USA). Each qRT-PCR analysis was performed in triplicate. Amplification efficiencies were calculated as 10 ^(−1/slope)^ – 1. Slope is the value obtained from the standard curve. The transcript quantifications of the candidate genes were performed in relation to *Actin* and calculated by 2^−△△CT^ method.

### Statistical analysis

Data were analyzed using a SPSS program (SPSS, Chicago, IL, USA). The differences among treatment means were evaluated by Duncan’s multiple range test at a 0.05 probability level. Differences between treatments and controls were evaluated by Student’s *t*-test.

## Results

### Morphology of the rudimentary leaves after ROS or NO treatment

**Fig. 1:**
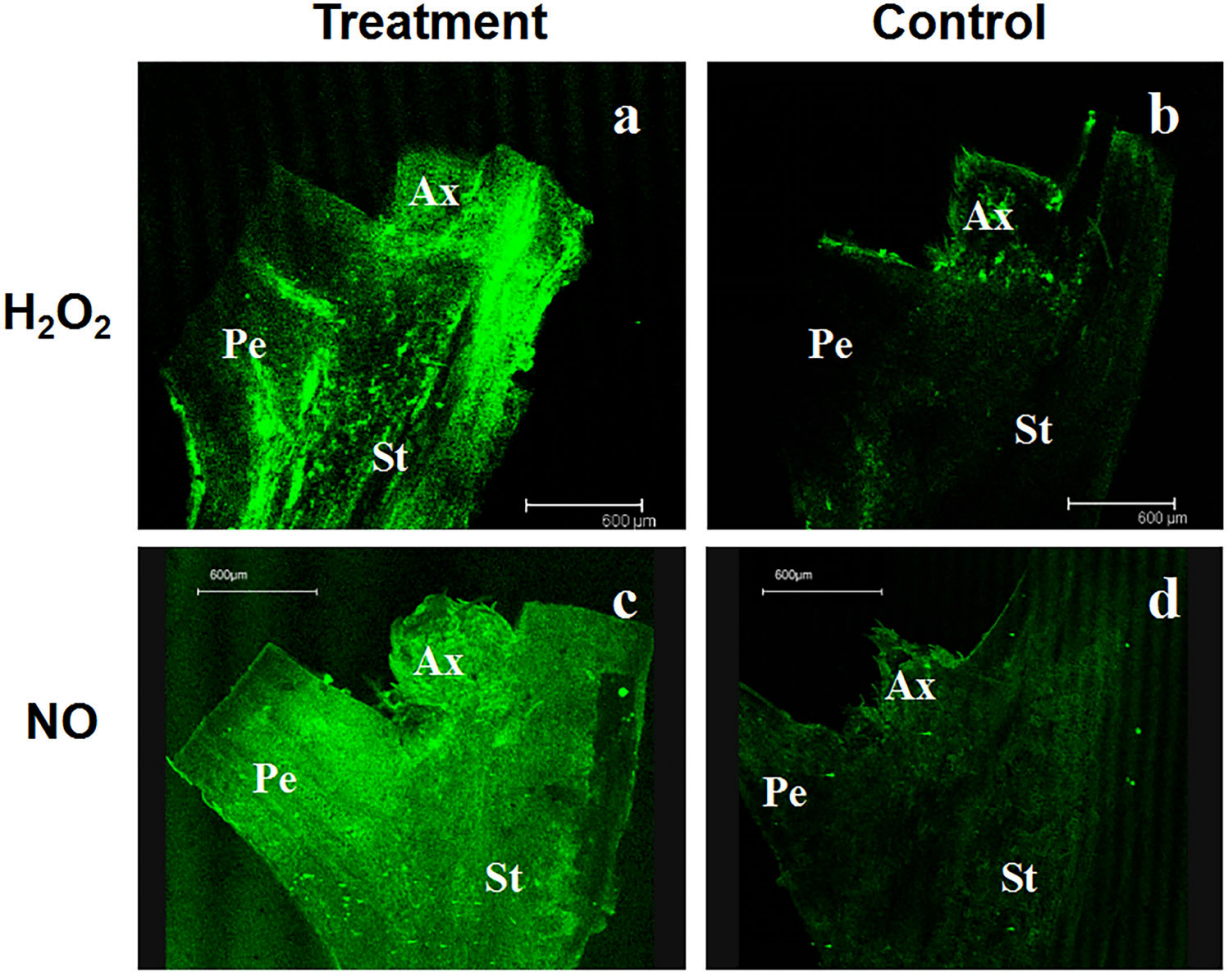
ROS and NO imaging under MV and SNP treatments. **a** DCFH-DA fluorescence of the MV-treated tissue. **b** DCFH-DA fluorescence of the control tissues. **c** DAF-2DA fluorescence of the SNP-treated tissues. **d** DAF-2DA fluorescence of the control tissues. A small piece of tissue after 10 h of treatment including stem and petiole bases were excised, dissected lengthwise, and incubated in DCFH-DA buffer, a ROS fluorescent probe, or NO indicator dye DAF-2DA. ROS or NO imaging was observed under confocal microscopy. Bar = 600 μm. Ax axillary bud, Pe petiole, St stem

**Fig. 2:**
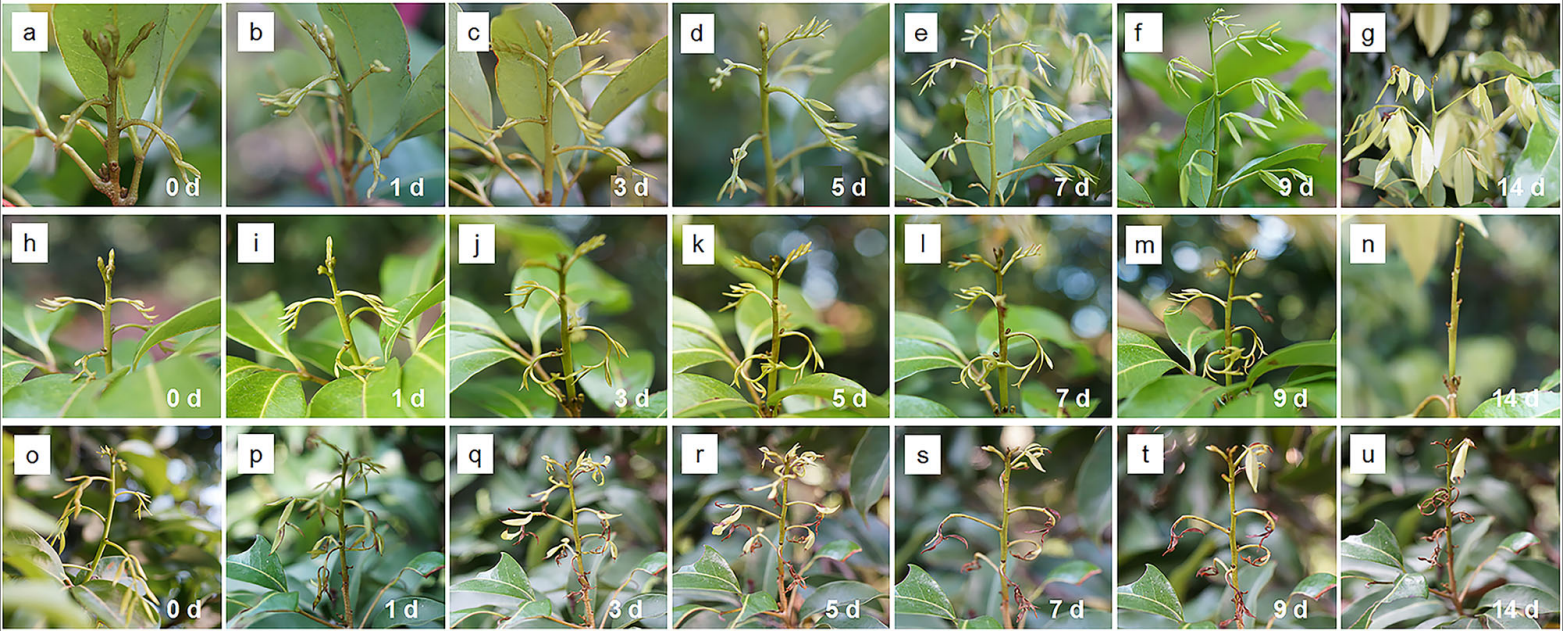
Phenotype of the ROS- and NO-treated rudimentary leaves in ‘Nuomici’ litchi trees. Branches of the ‘Nuomici’ litchi trees were uniformly sprayed with water as control, 120 µM MV as ROS treatment, or 3 mM SNP as NO treatment. **a**-**g** Control rudimentary leaves. **h**-**n** Rudimentary leaves after 0, 1, 3, 5, 7, 9, and 14 d of ROS treatment. **o**-**u** Rudimentary leaves after 0, 1, 3, 5, 7, 9, and 14 d of NO treatment

To determine whether the ROS inducer MV and the NO donor SNP work on the litchi rudimentary leaves, a ROS fluorescent probe DCFH-DA and a NO indicator dye DAF-2DA were used for ROS and NO imaging. As shown in Fig. 1, MV and NO-induced ROS and NO accumulation in the rudimentary leaves. Then morphological changes of the rudimentary leaves by MV and SNP treatment were investigated. It was shown that ROS and NO inhibited the growth of rudimentary leaves at an early developmental stage. The rudimentary leaves did not expand after spraying with the ROS inducer MV and the NO donor SNP, whereas the control rudimentary leaves continued to develop into compound leaves. After 3 d of ROS treatment and 1 d of NO treatment, the rudimentary leaves showed epinasty as characterized by downward curvature of leaves, presenting an early sign of abortion.^3^ At 14 d after treatment, the ROS-treated rudimentary leaves abscised, whereas the NO-treated ones showed wilting followed by abscission in response to a gentle touch (Fig. 2).

### Microstructure of the rudimentary leaves post ROS and NO treatment

**Fig. 3:**
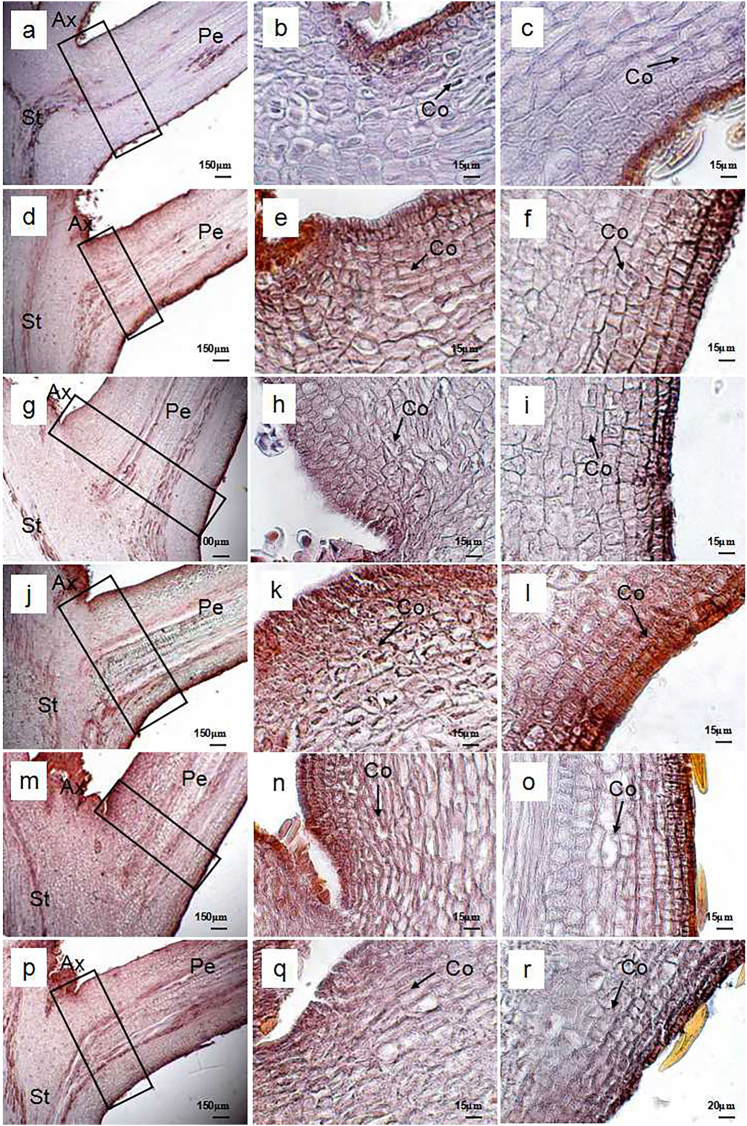
Light micrographs of petiole in the control rudimentary leaves of ‘Nuomici’ litchi. Leaf petioles were collected at 0 d **a**-**c**, 1 d **d**-**f**, 3 d **g**-**i**, 5 d **j**-**l**, 7 d **m**-**o**, and 9 d **p**-**r**after water treatment (control). Micrographs in the second column are enlarged images of the upper part of the black rectangular regions marked in the same line of the first column. Micrographs in the third column are enlarged images of the lower part of the black rectangular regions marked in the same line of the first column. Arrows are longitudinal splitting cortical cells. Ax axillary bud, Co cortex, Pe petiole, St stem

**Fig. 4:**
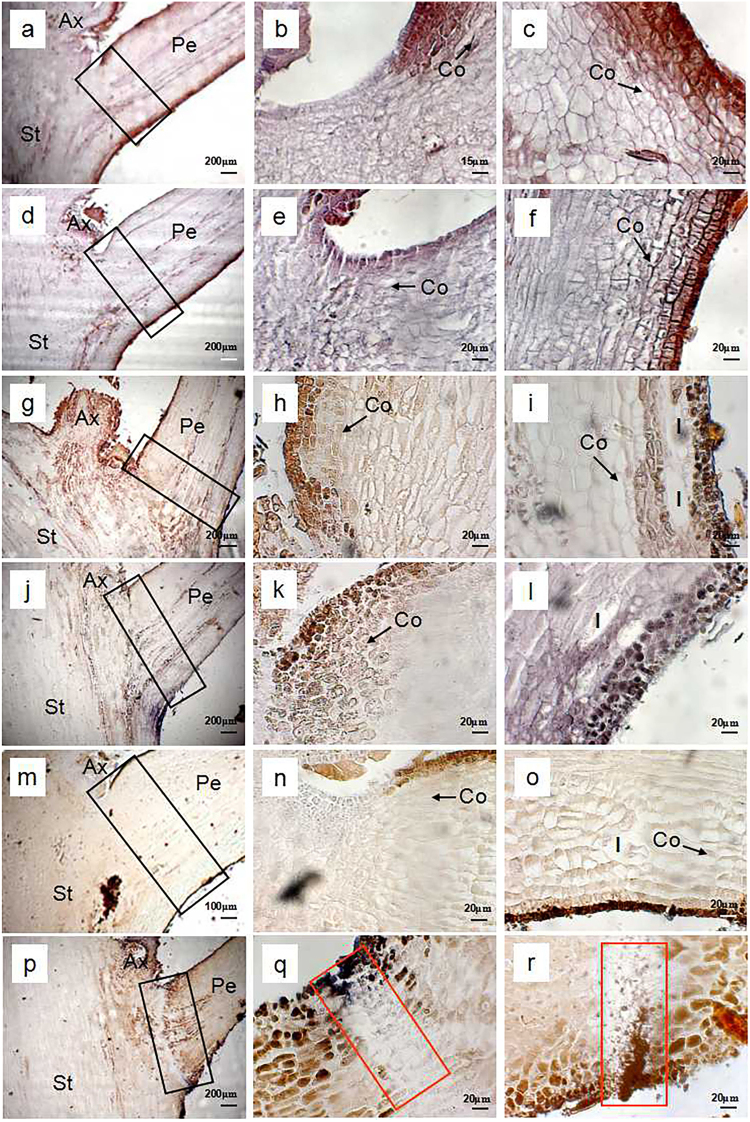
Light micrographs of petioles in the ROS-treated rudimentary leaves of ‘Nuomici’ litchi. The leaf petioles were collected in 0 d **a**-**c**, 1 d **d**-**f**, 3 d **g**-**i**, 5 d **j**-**l**, 7 d **m**-**o**, and 9 d **p**-**r** after methyl viologen dichloride hydrate (MV) treatment. Micrographs in the second column are enlarged images of the upper part of the black rectangular regions marked in the same line of the first column. Micrographs in the third column are enlarged images of the lower part of the black rectangular regions marked in the same line of the first column. Black arrows are longitudinal splitting cortical cells. Red rectangular regions are cell separation area in AZ. Ax axillary bud, Co cortex, Sc swollen cell, Pe petiole, St stem

**Fig. 5:**
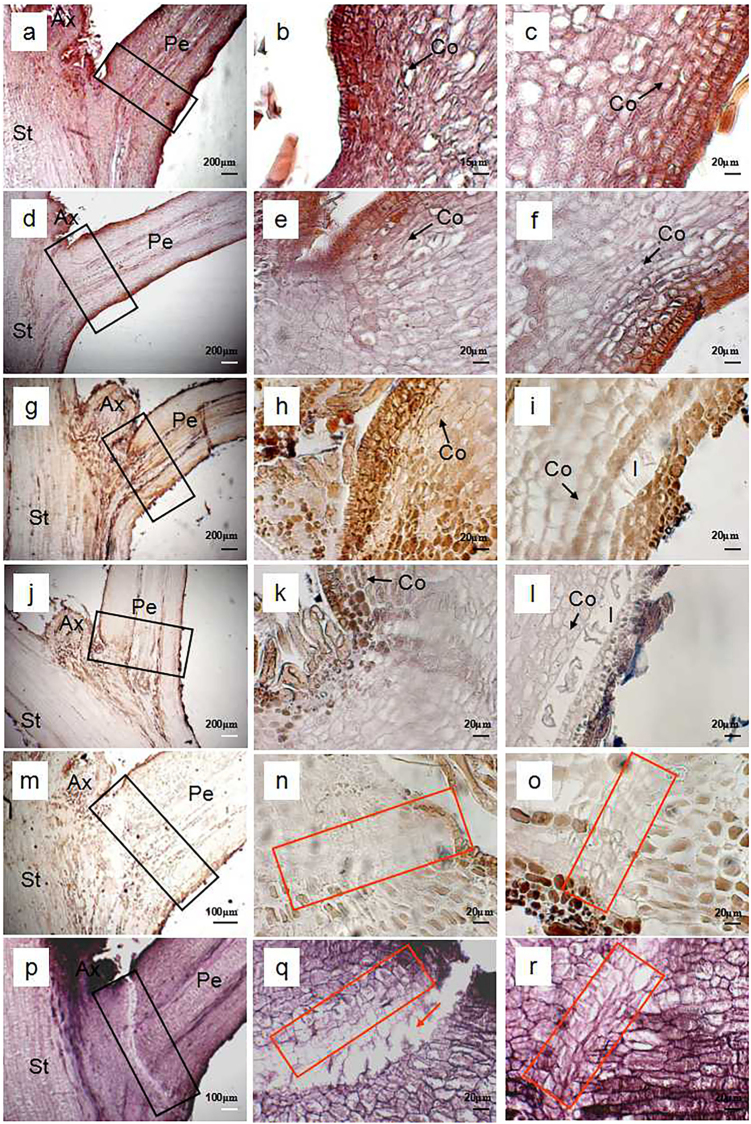
Light micrographs of petioles in the NO-treated rudimentary leaves of ‘Nuomici’ litchi. The leaf petioles were collected in 0 d **a**-**c**, 1 d **d**-**f**, 3 **g**-**i**, 5 **j**-**l**, 7 **m**-**o**, and 9 d **p**-**r** after sodium nitroprusside (SNP) treatment. Micrographs in the second column are enlarged images of the upper part of the black rectangular regions marked in the same line of the first column. Micrographs in the third column are enlarged images of the lower part of the black rectangular regions marked in the same line of the first column. Black arrows are longitudinal splitting cortical cells. Red rectangular regions are cell separation area in AZ. Ax axillary bud, Co cortex, Sc swollen cell, Pe petiole, St stem

Figures 3, 4, and 5 show the time courses of microstructure in the petiole of the rudimentary leaves after ROS or NO treatment. Cross section through potential abscission zone (AZ) of the control rudimentary leaves showed that cell morphology was the same as that of the adjacent region. Cell division was found in the cortex (Fig. 3). At 0 d and 1 d after treatment, no difference was found among the control (Figs. 3 a-f), ROS-treated cells (Figs. 4 a-f), and NO-treated cells (Figs. 5 a-f). However, from 3 d after treatment, changes in cell morphology were found among the treatments, particularly in the regions of AZ (Figs. 3, 4, and 5g-o). Both ROS- and NO-treated cells developed swollen cells in AZ or near the AZ from 3 d after treatment, however, their abscission mode was slightly different. In the ROS-treated leaves, swollen cells were found between 3 and 7 d (Figs. 4 i, l, o), and the rupture along the cell layer occurred at 9 d of ROS treatment (Figs. 4 p-r). Meanwhile, swollen cells in the NO-treated leaves were observed only between 3 and 5 d after treatment (Figs. 5 i, l). Several layers of flattened cells were formed in the AZ after 7 d of treatment, and then a distinguished boundary considered as an abscission layer was identified in the AZ (Figs. 5 m-o). Similarly to ROS-treated leaves, rupture occurred at 9 d after NO treatment (Figs. 5 p, q). In both ROS- and NO-treated leaves, longitudinal splitting of cortical cells were found before rupture occurred (Figs. 4 b, c, e, f, h, i, k, l, n, o; and Figs. 5b, c, e, f, h, i, k, l). Such cells were still observed at 7 d after treatment in the ROS-treated leaves (Figs. 4 n, o) but not in the NO-treated leaves due to the rupture (Figs. 5 n, o, q, r). Contrarily, those longitudinal splitting of cortical cells were observed in the control leaves, from 0 d to 9 d after treatment (Figs. 3 b, c, e, f, h, i, k, l, n, o, q, r).

### Ultrastructure of the rudimentary leaves post ROS and NO treatment

**Fig. 6:**
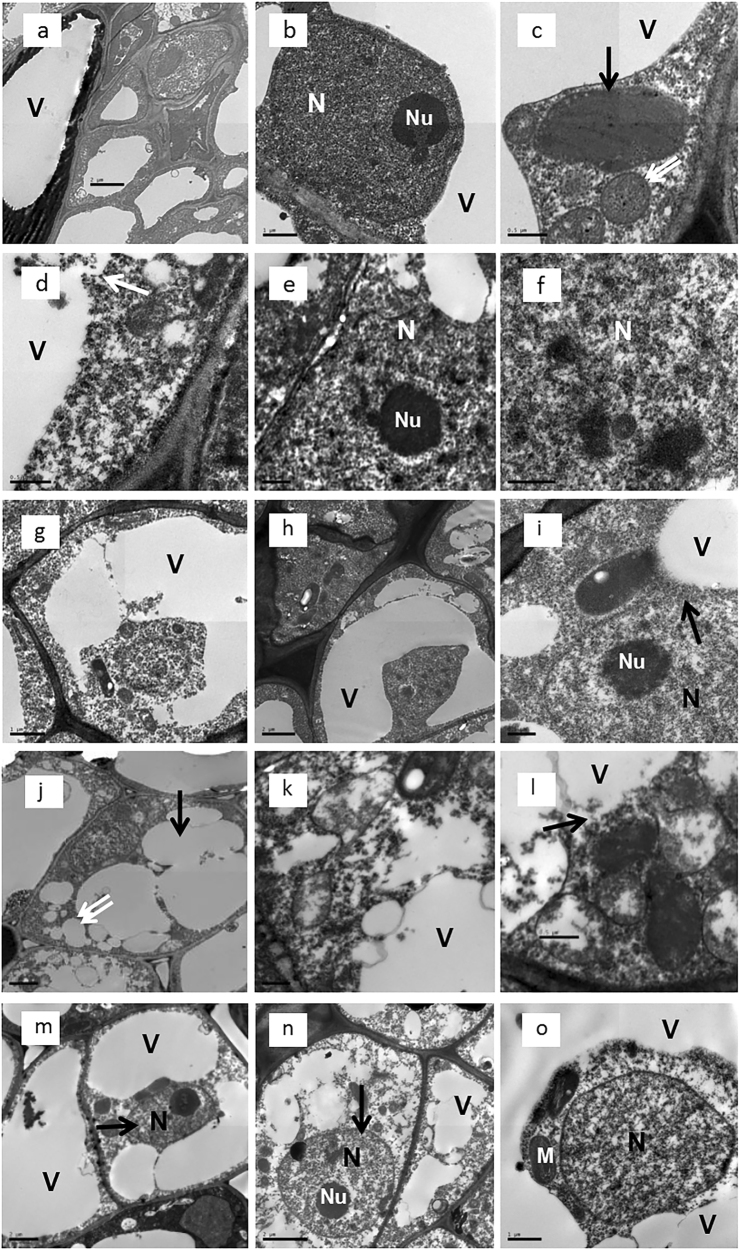
Ultrastructure of the petioles in the ROS- and NO-treated rudimentary leaves. **a**-**c** Cells before ROS or NO treatment: **a** cells exhibiting normal structure with clear tonoplasts, bar = 2 μm, **b** a nucleus with dispersed chromatin and clear nucleolus, bar = 1 μm, and **c** a mitochondrion (double arrows) and a chloroplast (arrow) with complete structure, bar = 0.5 μm. **d**-**f** Cells after 6 d of ROS treatment: **d** a cell exhibiting disrupted tonoplasts (arrow) and contents of the vacuole releasing into the cytoplasm, bar = 0.5 μm, **e** condensed chromatin, bar = 0.5 μm, and **f **a dispersed nucleolus, bar = 0.5 μm. **g**-**i** Cells after 9 d of ROS treatment: **g** a cell with disrupted tonoplasts and leakage vacuoles, bar = 1 μm, **h** cells showing a nucleus and part of the cytoplasm were surrounded by vacuoles, bar = 2 μm. **i** A cell showing disrupted nuclear membrane (arrow), bar = 0.5 μm. **j**-**l** Cells after 2 d of NO treatment: **j**-**l** cells after 2 d of NO treatment: **j** cells showing shrinking vacuoles (arrow) and cytoplasm with many vesicles (double arrows), bar = 5 μm, **k** a cell with contents of the vacuoles are releasing into the cytoplasm, bar = 2 μm, **l** disrupted tonoplasts (arrow), bar = 0.5 μm. **m**-**o** Cells after 5 d of NO treatment: **m** a cell showing its nucleus (arrow) was surrounded by vacuoles, bar = 2 μm, **n** a nucleus (arrow) with condensed chromatin, bar = 2 μm, and **o** a nucleus of which nucleolus disappeared, bar = 1 μm. M mitochondrion, N nucleus, Nu nucleolus, V vacuole

Based on ultrastructural analysis of the nonvascular tissue in the petiole base of the rudimentary leaves, all the cells exhibited normal structure with consecutive tonoplasts before treatment (Fig. 6a). The normal cell had a nucleus with dispersed chromatin and clear nucleoli (Fig. 6b). Structure of the chloroplast was well developed, and the mitochondria were intact with richly invaginated internal membranes (Fig. 6c). However, tonoplasts of the cells were disrupted at 6 d after ROS treatment, and contents of the vacuole were released into the cytoplasm showing early signs of cell autolysis (Fig. 6d). Moreover, chromatin of the cells were condensed, and nucleoli were dispersed (Figs. 6e, f). At 9 d after ROS treatment, tonoplasts of the cells were also disrupted as those of the cells after 6 d of treatment. Cytoplasm of the cells was leaked into the vacuoles (Fig. 6g). Interestingly, it was found that nuclei and part of the cytoplasm were surrounded by enlarged vacuoles (Figs. 6g, h). Nuclear membranes were disrupted and the nucleoplasm was leaked into the cytoplasm (Fig. 6i).

Similar cellular events were observed in NO-treated cells. Figures 6j-l show the ultrastructure of the petiole base of the rudimentary leaves after 2 d of NO treatment. Some vacuoles of the cells became shrunk and many vesicles were scattered in the cytoplasm (Fig. 6j). Contents of the vacuole were released into the cytoplasm showing cell autolysis (Fig. 6k). The tonoplasts of the cells were disrupted and cytoplasm was leaked into the vacuoles (Fig. 6l). Chromatin was condensed (Fig. 6n), and nucleolus disappeared in some of the NO-treated cells (Fig. 6o). At 5 d after treatment, nuclei surrounded by enlarged vacuoles were also found in the cells of the NO-treated leaves (Figs. 6m, o).

### Morphology of the nuclei and percentage of PCD in the ROS- and NO-treated rudimentary leaves

**Fig. 7:**
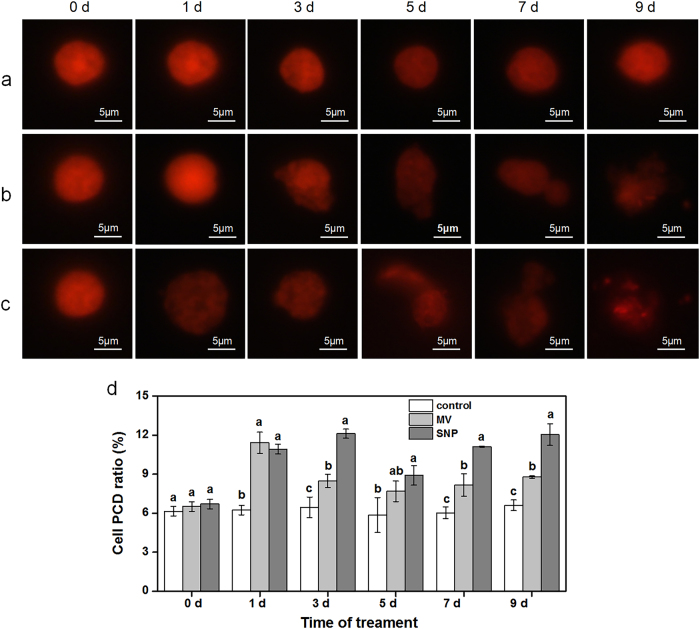
Images of **a** the nuclei in the control cells, **b** the ROS-treated cells, **c **the NO-treated cells, and **d** cell PCD ratio of the rudimentary after ROS and NO treatments. Branches of the ‘Nuomici’ litchi trees were uniformly sprayed with water as control, 120 µM methyl viologen dichloride hydrate (MV) as ROS treatment, or 3 mM sodium nitroprusside (SNP) as NO treatment. Rudimentary leaves were collected after 0, 1, 3, 5, 7, and 9 d of treatment. Nuclei images were visualized by fluorescence microscopy. Bar = 5 μm. Cell PCD ratio was given by the expression 100× numbers of PCD nuclei/total numbers of nuclei scanned. Values are means ± SE, *n* = 3. Different letters indicate significant difference at *P* < 0.05 level among treatments at the same time according to Duncan’s multiple range test

Figures 7a-c show the morphology of the nuclei stained by PI. At 0 d after treatment, all the nuclei were clear in shape with high fluorescence intensity. At 1 d after treatment, the nuclei in the ROS-treated cells were still as clear as those in the control cells. However, those of the NO-treated cells fluoresced less than the control. After 3 d time point and from then on, the nuclei in the cells treated with either ROS or NO fluoresced less than the control. Furthermore, the nuclei shape of those ROS- and NO-treated cells became irregular. These cellular events occurred in a different manner between ROS- and NO- treated cells at 9 d after treatment. The nuclei in the ROS-treated cells become more irregular and had the tendency to disrupt, whereas those in the NO-treated cells disrupted into fragments.

To analyze the cell PCD ration, a FCM was used by PI labeling. At 0-d time point, all the PCD ratios of the control, the ROS- and NO-treated leaves were the same as shown in Fig. 7d and Supplementary Figure S1. However, at 1 d after treatment and from then on, the PCD ratios of ROS- and NO-treated leaves were significantly higher than those of the control except for the ROS-treated leaves at 5-d time point. For example, the PCD ratios of the ROS- and NO-treated leaves were significantly increased by 34.5% and 90.9% at 3-d time point. These results suggested that cell PCD ratio increased by ROS and NO treatments from the first day of exposure to these compounds.

### Expression of the senescence-related genes in rudimentary leaves after ROS or NO treatment

**Fig. 8:**
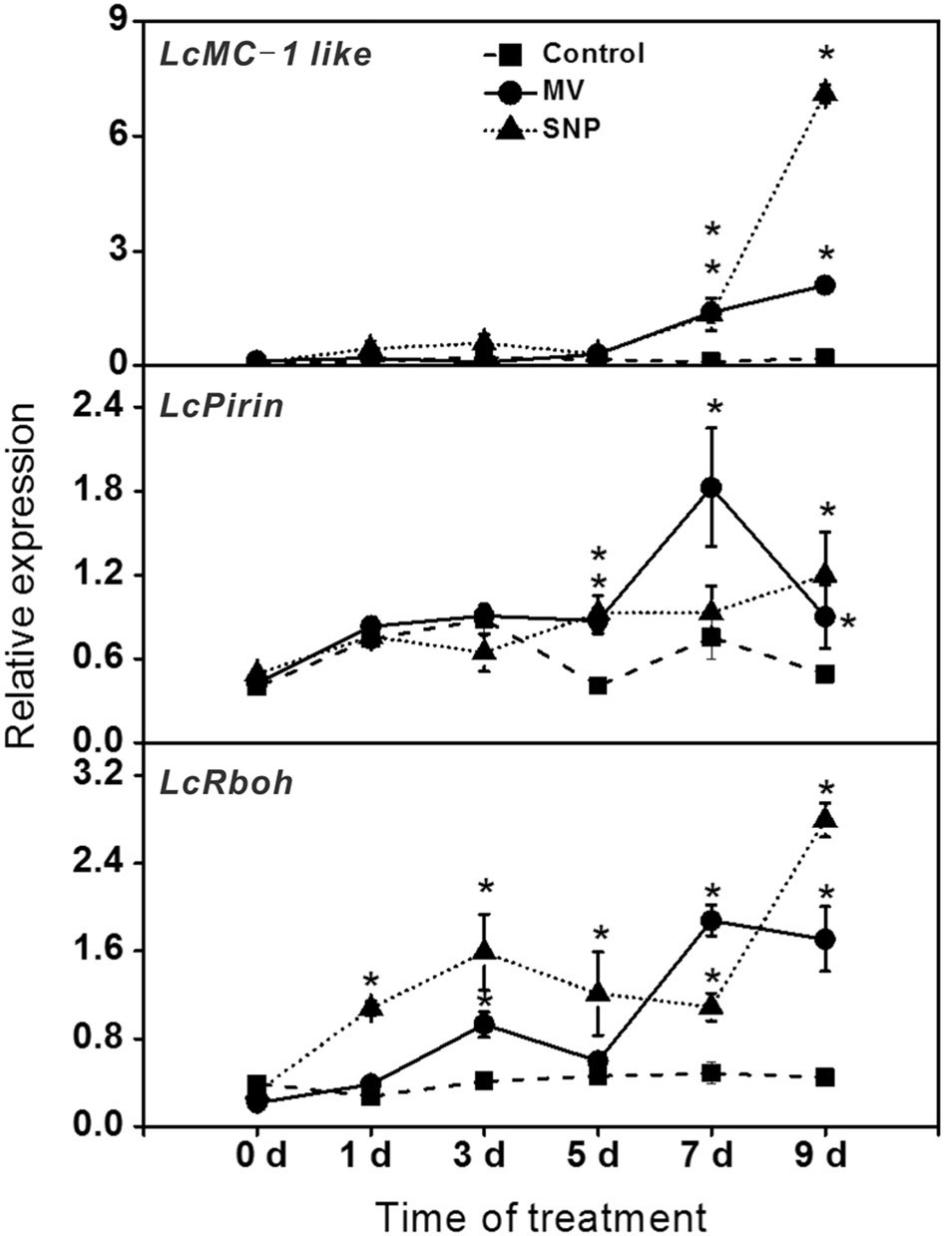
Time course of the relative quantities of *LcMC-1-like*, *LcPirin*, and *LcRboh* in the rudimentary leaves after ROS or NO treatment. Branches of the ‘Nuomici’ litchi trees were uniformly sprayed with water as control, 120 µM methyl viologen dichloride hydrate (MV) as ROS treatment, or 3 mM sodium nitroprusside (SNP) as NO treatment. Rudimentary leaves were collected for determination of gene expression after 0, 1, 3, 5, 7, and 9 d of treatment. Relative transcription was calculated by RT-qPCR using the 2^−ΔΔCT^ method with actin as reference gene. Data are means of three replicates and the bars represent SE. Significant differences (*P* < 0.05) according to the Student’s* t-*test between the treatment and the control are indicated by the asterisks

To identify genes involved in the ROS or NO-induced senescence and abscission of the rudimentary leaves, we first screened the ROS-responsive senescence-associated genes (SAGs) from our RNA-seq data set of the ROS-treated rudimentary leaves,^19^ and identified 136 SAGs from the differentially expressed gene data set (Supplementary Table 2). We then selected eight PCD-related genes as candidate genes. The sequences of the candidate genes are shown in Supplementary File 1. These PCD-related genes were *LcMC-1-like, LcBAD, LcDAD-1, LcBI-1, LcS-like, LcPirin, LcWIP, and LcRboh*. They were subjected to qRT-PCR analysis for further screening. According to the results (Supplementary Figure S2), we selected those genes whose relative expression levels increased after ROS or NO treatment. Relative expression of those genes was then determined under a time course of ROS or NO treatment. As shown in Fig. 8, relative expression of *LcMC-1-like* in the ROS and NO-treated rudimentary leaves significantly increased at 7 d and 9 d after treatment. For example, at 9-d time point, the expression levels were 932% higher in the ROS-treated leaves and 3390% higher in the NO-treated ones than the control. Relative expression of *LcPirin* in the ROS and NO-treated rudimentary leaves increased after treatment. At 5 and 9 d after ROS or NO treatment, relative expression levels in the ROS- and NO-treated leaves were significantly higher than those of the control. As to *LcRboh*, relative expression level also showed an increasing trend after ROS or NO treatment. Particularly, increased *LcRboh* expression was found at an early stage after treatment, by 4 d earlier than those of *LcPirin* and *LcMC-1-like*. At 3-d time point, relative expression level of the *LcRboh* was the highest in the NO-treated ones (285% increase), followed by the ROS-treated leaves (126% increase) compared with that in the control.

## Discussion

Rapid cell responses to environmental stress confers growth and reproductive advantages by ensuring the survival of the species^4^. ROS and NO are key signaling molecules involved in plant responses to an environmental stress^7,8^. We previously found that ROS and NO promoted flowering partially by inhibiting the growth of panicle rudimentary leaves^10^. Hence, the understanding how ROS and NO affect the growth of rudimentary leaves is of great importance to control flowering in litchi.

In the present study, we found that ROS or NO treatment inhibited the growth of the rudimentary leaves at an early stage and led to leaf abscission a later stage. This suggests that ROS and NO are important mediators in inducing senescence and abscission of the rudimentary leaves in litchi. Regardless of the treatments, it took about 14 d from the commencement of cellular responses until the completion of the entire process. This observation indicates that such slow process is the result of a protracted abscission induced by ROS and NO treatment rather than rapid abscission. This research was conducted in early winter condition when the temperature ranged from 16 to 20 °C. In other plant systems, such as Azolla, a small water fern, there are two types of abscission: an ethylene-induced protracted abscission and a rapid abscission occurring within 20 min in response to sodium azide or transient exposure to high-temperature stress^25,26^. Interestingly, we found that petioles of the rudimentary leaves displayed downward growth after ROS or NO treatment (Fig. 2)^3^, which is characterized as, our previous studies showed increased expression levels of many genes encoding ethylene signal transduction components after ROS treatment^19^. Hence, we suggest that ethylene may be involved in the protracted abscission of the rudimentary leaves after the treatment with ROS or NO. Further investigation should be carried out to elucidate the role of ethylene on the ROS- and NO-induced abscission of the rudimentary leaves in litchi.

Abscission is the last stage of senescence by which plants shed unnecessary organs. Organ abscission occurs at a specialized region known as the AZ. The AZ consists of several layers of microscopic cells that are distinct from the surrounding cells and formed well before organ separation occurs^27^. In the present studies, we found that rupture occurred in the AZ of rudimentary leaves after 9 d of treatment with either ROS or NO (Figs. 4 and 5), suggesting that the abscission underwent normal abscission procedure, a protracted abscission. Interestingly, swollen cells were found in the AZ or near the AZ of the ROS- or NO-treated rudimentary leaves (Figs. 4 and 5). Formation of the swollen cells might be related to the decrease in the osmotic potential of cells in the AZ and be beneficial to the rupture of the vascular bundle^28,29^.

Observation of ultrastructure of cells by electron microscopy and nuclei stained by PI under fluorescence microscopy revealed that the ROS- or NO-treated cells showed irregular shape of the nuclei, chromatin condensation, disappearance of nucleolus and even nuclear fragmentation (Figs. 6 and 7), which are reported as the PCD hallmarks^30,31^. These results show the evidence that PCD may be involved in the ROS and NO-induced senescence and abscission in the rudimentary leaves. We also found disrupted tonoplasts in both the ROS- and NO-treated cells and formation of vesicles within cytoplasm in the NO-treated cells. These results indicate that infusion of the cytoplasm and the vacuoles occurred, leading to cell autolysis. Interestingly, it was found that nuclei and part of the cytoplasm were surrounded by enlarged vacuoles at later stages after treatment with ROS and NO (Figs. 6, g, h, m, o), showing increased volume of the vacuoles and marked shrinkage of the cytoplasm. These features exhibited in the ROS- and NO-treated cells were consistent with those in the Norway spruce embryo-suspensor cells, which were the features of the vacuolar PCD^31^. Vacuolar cell death is often manifested by a gradual decrease in the volume of the cytoplasm, a concomitant increase in the volume occupied by vacuoles, and an engulfment of the cytoplasm by vacuoles with subsequent degradation^32^. As our electron micrographs manifested those vacuolar PCD features, we suggest that vacuolar PCD may be involved in the ROS- and NO-induced senescence and abscission of the rudimentary leaves in litchi.

PI flow cytometric assay has been widely used for the evaluation of apoptosis in different tissues^33^. Apoptotic nuclei appear as a broad hypodiploxd DNA peak, which was easily discriminable from the narrow peak of normal (diploid) DNA content in the red fluorescence channels as PI is capable of binding and labeling DNA^34–36^. FCM measurements was proven to be a quantitative technology to study PCD dynamics^37^. In this study, ROS or NO treatment significantly increased the percentage of PCD in the rudimentary leaves (Fig. 7d), further supporting the PCD involvement in the ROS- and NO-induced senescence and abscission of the rudimentary leaves in litchi.

Induction of different protease activities has been associated with the occurrence of senescence^38,39^. Caspase like is involved in protein degradation^40^. *Lepirin*-deduced protein is homologous to the human protein Pirin, a nuclear factor reported to form quaternary complexes with the transcription factors NF-κB and Bcl-3, and target sequences in the promoter regions of anti-apoptotic genes. ROS are key components in plant cell death associated with developmental processes and environmental stress responses^41^. Nicotinamide adenine dinucleotide phosphate oxidase whose subunit is encoded by *Rboh*, is a key enzyme for ROS production in plants in response to biotic and abiotic stresses and during PCD^31,42^. Our results showed that the litchi Caspase-like homolog *LcMC-1-like*, *LcPirin*, and *LcRboh* were significantly induced after ROS or NO treatment (Fig. 8), and their expression levels increased with the progression of senescence, suggesting that these genes may be involved in the ROS and NO-induced senescence and abscission of the rudimentary leaves in litchi. However, the induction of these genes did not occur simultaneously. *LcRboh* was induced the earliest and *LcMC-1-like* the latest (Fig. 8), suggesting that they may play different roles in the senescence process. Further study should be carried out to investigate their gene function in the senescence in litchi rudimentary leaves.

## Conclusions

ROS and NO-induced senescence of the rudimentary leaves in litchi. PCD may be involved in the ROS- and NO-induced senescence of the rudimentary leaves. *LcMC-1-like*, *LcPirin*, and *LcRboh* were significantly induced after ROS or NO treatment, and their expression levels increased with the progression of the ROS- and NO-induced leaf senescence.

## Electronic supplementary material

Supplementary Figure S1(DOC 380 kb)

Supplementary Figure S2(DOC 74 kb)

Supplementary Table 1(DOC 53 kb)

Supplementary File 1(DOCX 18 kb)

Supplementary Table 2(XLS 74 kb)
