# Supplementary material for: Reactive oxygen species and nitric oxide induce senescence of rudimentary leaves and the expression profiles of the related genes in Litchi chinensis
Source: Hortic Res. 2018 May 1;5:23. doi: 10.1038/s41438-018-0029-y (PMC5928110; doi:10.1038/s41438-018-0029-y)
Supplement: Supplementary file 1 — Supplementary Figure S1(DOC 380 kb) [file 41438_2018_29_MOESM1_ESM.doc]

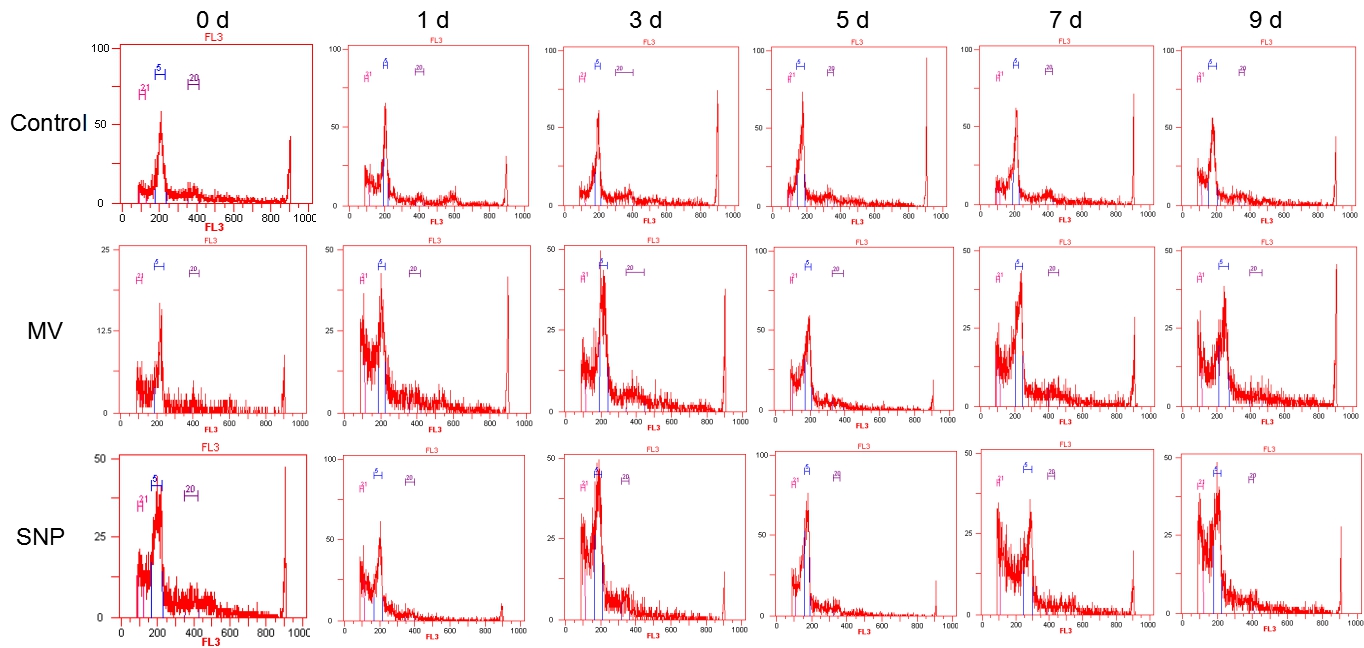


Supplementary Figure S1 Determination of percentage of PCD in the control, MV, and SNP treated rudimentary leaves by flow cytometer. Branches of the ‘Nuomici’ litchi trees were uniformly sprayed with water as control, 120 µM MV as ROS treatment, or 3 mM SNP as NO treatment. Rudimentary leaves were collected for determination of the percentage of PCD by flow cytometer after 0 d to 9 d of treatment. FL3, the fluorescence channel; Region 5, nuclear amount of normal cells; Region 20, nuclear amount of mitotic cells; Region 21, PCD cells.
