# Supplementary material for: Reactive oxygen species and nitric oxide induce senescence of rudimentary leaves and the expression profiles of the related genes in Litchi chinensis
Source: Hortic Res. 2018 May 1;5:23. doi: 10.1038/s41438-018-0029-y (PMC5928110; doi:10.1038/s41438-018-0029-y)
Supplement: Supplementary file 2 — Supplementary Figure S2(DOC 74 kb) [file 41438_2018_29_MOESM2_ESM.doc]

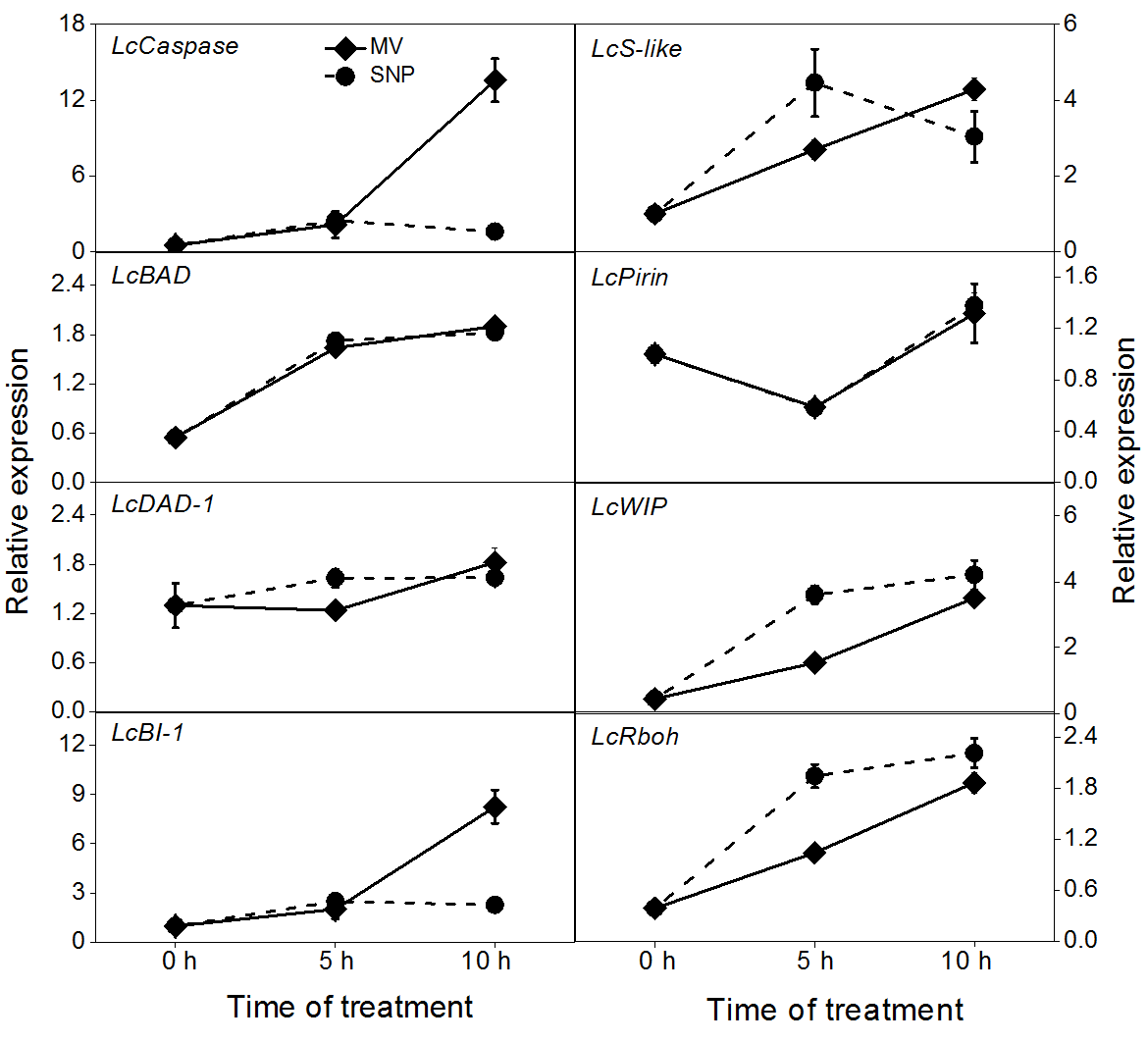


*LcMC-1 like*

Supplementary Figure S2 Time course of the relative quantities of the candidate genes in the rudimentary leaves after ROS or NO treatment. About 6 cm length of branches with new flushes were cut off from the trees and immediately placed in water or solutions containing 40 µM MV or 0.5 mM SNP. All the cuttings were placed in a growth chamber at 160 μmol·m^-2^·s^-1^ photosynthetic photon flux density at 20℃. After 0 h, 5 h and 10 h, the third and the fourth rudimentary leaves were sampled for determination of gene expression. Relative transcription was calculated by qRT-PCR using the 2^-ΔΔCT^ method with actin as reference gene. Data are means of three replicates and the bars represent SE.
