# Supplementary material for: Reactive oxygen species and nitric oxide induce senescence of rudimentary leaves and the expression profiles of the related genes in Litchi chinensis
Source: Hortic Res. 2018 May 1;5:23. doi: 10.1038/s41438-018-0029-y (PMC5928110; doi:10.1038/s41438-018-0029-y)
Supplement: Supplementary file 3 — Supplementary Table 1(DOC 53 kb) [file 41438_2018_29_MOESM3_ESM.doc]

Supplementary Table 1 Primer sequences of senescence related genes for qRT-PCR.

| gene | Unigene ID | Forward primer (5'→3') | Reverse primer (5'→3') |
| --- | --- | --- | --- |
| *β-actin* |  | AGTTTGGTTGATGTGGGAGAC | TGGCTGAACCCGAGATGAT |
| *LcCaspase* | Unigene0064448 | CACAGCGTTTCGTCATAGCC | GTTGAGAGGTTGGGGTTTCC |
| *LcBAD* | Unigene0034267 | TCTCGCTACTCTGGTCTT | ATCCTCAGGGGCATCT |
| *LcBI-1* | Unigene0020640 | TGGAAGGAGTTCATCG | CTCGTGCCTCACAAGT |
| *LcDAD-1* | Unigene0017482 | CTTGTGTTGGGACAGCAGTT | CTACCGTGTTCTACATTTCA |
| *LcPirin* | Unigene0079499 | TCATCCACATAGAGGGTT | TTGTTCTGCTGGCATTTC |
| *LcS-like* | Unigene0031244 | TGACCAGAACTTAGAGCCATTA | ACTGTGACACGCAGCAAA |
| *Lc**WIP* | Unigene0007171 | TCCTCGTCCCAGTTGTAG | ACCACTTGTCGGTTCTTG |
| *LcRboh* | Unigene0045124 | AAAGGGTATGGCAAGATG | AGGATTGAGATGGTGGAG |
