# Supplementary material for: Reactive oxygen species and nitric oxide induce senescence of rudimentary leaves and the expression profiles of the related genes in Litchi chinensis
Source: Hortic Res. 2018 May 1;5:23. doi: 10.1038/s41438-018-0029-y (PMC5928110; doi:10.1038/s41438-018-0029-y)
Supplement: Supplementary file 4 — Supplementary File 1(DOCX 18 kb) [file 41438_2018_29_MOESM4_ESM.docx]

Sequences of senescence related genes

*LcMC-1 like*

>Unigene0064448

GGCCGGGTTCATTTTCCAGTGCTTGAATGAAGCTGATTGTCAACGCACCCGTCGATAGTTTGTCTATAAACCCAGTGCTATCAGATGAGTTTTCATTGTCATCACAAGCACTTATAGAGAGAGCTAGCCCGCCGCTTGTACCTTTGTAACAAGCCGCTGGAAGTCGTTGATCTTCCCACCTATAGTAACCTTCGCCGTTCATCCTACAGACAAATGGTAGGTCGAGTATAGTTCCACTACGGCAGGAATCAACAATGGCATGAAGTTTGACTCCATAAGTCAATGGCCTTACAATTGTTTCATTGATTTCATCATCAATTATGGGACCCTCGGTTTCATAATCAAGAGGACACAGCGTTTCGTCATAGCCATCTACCTCATCATTGTTATAGTCACGCTGCCTTGAACCATGCCCCGAGAAATGAAACACCAGCGAGTCCCCCGGCTGGCAACCTTGAACCAGCCATCGCATGGCTGTTCGGATGTTTTGTTTTGTGGGAATCCTAAGTGGATTATTCTCTTCATCTGTTAGCATGAGGATGCAGTCAACAGGAAACCCCAACCTCTCAACGAGAAGATACTTCATGCACTTAACATCGTTGACACTTCCTTTAAGGCTGTAACTTTTTTGACGATAATTCACTCCACAAAGAAGAGCCCTTTTCCTTCCATAAACTGGAGGAGAAGGCAAAGCAGGACGTAGAGGACGCG

*Lcpirin*

>Unigene0079499

TTTGTTTGTTTTTGTTTTTGTTTTGGTTTTAGAAACCAAAAATGCCTGAGACGGAAATTGTGAGAGAACCAAGATTGGTGGTGAGGAAATTCCTTGCAAGGCCTCAACATGAAGGGCTTGGAGCCATTGTGAGAAGAAGCATAGGAAGGTTTGAGTTGAGATACTTTGATCCTTTTCTTGCTTTGGATGAATTTTCAGTCACTGCTCCAGCTGGGTTCCCTGATCATCCACATAGAGGGTTTGAGACAGTGACGTACATGCTACAGGGAGCGGTAACGCATGAGGATTTTGGAGGGCATAAGGGGACTATAAAAACTGGGGACTTACAATGGATGACTGCAGGAAGAGGGATTGTTCACTCAGAAATGCCAGCAGAACAAGGCACACAGAAGGGATTACAGCTGTGGATCAACCTCTCTTCCAAGCATAAAATGATTGACCCAAGGTATCAAGAAGTGACAAGTAAGGACATAGCAGAAGCTAGCAAAGATGGGATCAAAGTTAGAGTCATAGCAGGAGAAGCGTTGGGAGCCAAGTCACCAGTCTACACAAGAACACCAACAATGTACTTGGATTTCACCATGAAACCTGGAGCTCACCTCCAGCAACCGATCCCAAAATCATGGAATTCATTCGTATACGTCCTGGAAGGCGAAGGAATCTTTGGAGGGAAGAAGTCCTCGCCGGTGTCAGCCCACAACCTGCTCCTACTGGGGTCTGGGGATGGCCTGGAAGCATGGAACAAGTCCACCAAGCCCCTCAGGTTCATTTTGGTGGGTGGTGAACCATTGGGTGAGCCACTAGTGCAGTTTGGTCCATTTGTGATGAACACACAAGAAGAGATTGATCAAACAATTGAAGATTTTGAGAATTGTGTTAATGGATTTGAAAAAGCAAAGCATTGGAGATCAGAGGCTGCAATTAGCCTTGATTTTTAGATTATATTTTATTGGAGAAGGAGAATTTATTGGT

*LcRboh*

>Unigene0045124

TCACAACTACTGCACTAGTGTTTACGAAGAAGGTGACGCTAGGTCCGCGCTCATTGCAATGTTACAGTCCCTCAATCACGCCAAGAATGGTGTCGACATCGTGTCAGGCACGCGTGTCAAGTCCCATTTTGCCAAGCCCGATTGGAGGAACGTGTACAAGCGGATTGCTCTTAACCACACCGATACAAGAGTTGGGGTGTTTTATTGTGGGTCACCAGCACTGACAAAGGAGTTGCGCCAGCTGGCTCTGGATTTCTCTCACAAGACCACCACCAAGTTCGATTTCCATAAAGAGAACTTCTAATCTCAACGTTCCAATCACTGGAAGTGCCCACCAGCTAAAGTCCCTCCTATTTTCCTCTCCATTATTATATACGTGTAACTCCTCCTCACCTACAAAGATTCTGGCCGAGCTATGTTCATAGAATGTTAGAACGCTATCAATTGTTGATGTTTCATAATGACTTTATTATTGCTGAATGTTGGACAATCACTAATATAAAGTTAGGTACAGAATGCAGACAAGAAATGGTGGGTTTGTTTGATTTTTGGGTGACCCACGAACCAGAAGCCTTGGGAAGAAGTTGGAAAGAAATGAGGAAAAGGGTATGGCAAGATGTCACGCTCCTTCACACCTTTTCTGTTTGATAGCGGGGGTTCAATTCAATCAATTTCAGAGCAACTGTAAAGTGCCGGCCTCCACCATCTCAATCCTCAAGGAAAGAATATATATGTATTATTTTTCTTTTTTTAGAAGAAATGAATTTTTTTTTTTTTGTCAAAACTAAAGACTCAAAACTGTTACAAAATGCATATCCA

*LcWIP*

>Unigene0007171

GTTCATTTATCCGAAAAAGGAGGCTTTAGTTTAGGAAAAAACCAAAAATTGAAGGAGACTATAGCAAAACTCCCCACACAAAACACGCAGAAAGTGAAACGTGTAAATCACGAAACTACCATTCCATCGCTCTATATAATGCCCTCCTCGTCCCAGTTGTAGTCCTCACCAGTTTCTTCTGGTTCAAACAACACCTCACCTTTAGAAAATAAAAAACAAAACAAAACCCACTGTGCTGTCTCTGTTGTTTAAAGGTATTTTCAGACACGAACTGGCTAACTCTCAGGATAAAGCTCTGGATGAGACCGAACACAAGAACCGACAAGTGGTTCGTGCGCTTTATGACGCCTTGAAGTCACGTGACGTCCAGACCGTCCACAAGATTCTCGCTTCTGATCTTGAGTGGTGGTTCCATGGCCCGCCGACCCACCAGTTTATGATGCGCCTCCTCACTGGCGGTGCCTCCACGTCGTCAGCATTCGAGTTTGTTTTCACGCCCTCGCAGGTCCAGTCGTTCGGGCCCACCACCGTCGTCGCTGAGGGCTGCGATCACAGCCGTCAAATCTCCTGGGTCCACGCTTGGACGGTCACGGATGGGATAATCACCCAGGTGAGGGAATACTTCAACACGTCTCTCACTGTCACCCGCCTTGGACCGTCCGATTGCTCTCCGGAGTCTGCTTCATCGACGGTCGAGATTAATTCCGTGCATTGCCCTTCCGTTTGGGAGAGTAGCTCCTCTAACCTGTTCGGTAAATCGGTTCCGGGTTTGGTTCTCGCTATATAATACAATATCCGAACCCGAATATAAGGCAATCACGTGTTGGCTTGTAATTGGTATGCTTGTTTGTTCTAGAGAGAGAGAGAGAGAGAGAGAGTGAAAGACACAGTGTATGTTTGTGGTCTGGGAGTGACTGAGTGAGTTAGAGAGTAGAGTTACGTTTTGCTAAATAATAAAATGGAAGAGAAGAGAGGTTGTGGAGATCGAGATGGCGGGGGTGGGACCCACATGGCAGACAAATGACAATAACGAAAGACTGACCGGAGAAAACGTTGGGTGC

*LcS-like*

>Unigene0031244

ACATCACTTCCAGGGTTTTTGTTAAGGACTACCAAAAAACACACTTTCCGCAACTAATATCTGAACAACCTTTGAATGTCATCAAGGATTTGACTTAAACGATTAAAAGAGAAGGGTAACCTGAATTCTTAAGAATTTTGAGAAGGTCTATTTGGTCTTTGAGGTTGAGAACAGCTTCGAAGTACGCATGCTGGTCAAGGACACATTCCGAGCAGGTTCCATGTTTATTCCATTCATGTGACCAGAACTTAGAGCCATTACTACTTGGGCATGCAAGCGTTGGCCAGCTAATTTGCATTCTGCTAATTAGGTCTGAGATCTTGGATTCATTGTAAGGGTTGTGAGAATCACAGTATGATGGGTGTGAGCCACTGTTGTAAATGGGCCAAAGACCATGAATGCTGAAATTTGTCGCAGGTTTTCCAGCTGTTGGGTAGCAACAATTTTGCTGCGTGTCACAGTATGATCCTGGCCACTGATTTACTGGCTTCTCAATGCTGTGGAACTTGCTTGTATTCATTGGCGAATGAATTTTGATTCAAATACCAAGGCACATCTACACACGCCAAACCCATCTCTTCAAACTTCGAACCAACAAGCTAATGATATGCAACTTCTTAAATAAAACCTCAAAAAAGGATCGAAGCACTATAATCAACATTTTTTT

*LcBAD*

>Unigene0034267

GTGGACATGAATCGTAAATTGAAGCTAAACATGATTATCTCTTATTACATAAACAAGCTA

ATAAATCACTACTCCCATTTGGTGAACGATGTTCCTCCAATTCCGTATATGGGGGACATT

GAGGCAACCAAAGACACCTTTATTATCTATCTACCTAACATGGTATATTACATGGTGCAT

ACATGAAATATAGAAAACGAAAATGACATGCATGAAATGCAACATCTATTATGATTTTTT

CACAACTTGGGTGCTTGGATGAGCTAGTGTTCAAGTGTATCTCTCACCTGCTACCTATTCAATACATTTGCCTTTATACTTTTATTTGGTTTGGATGGGAGCAAATGTGTGATATTAGTCATTGCAGACACCAGTGATGGTAGACTATAAGGGGAAAGAAAGGATATTCTTGTTCAGGAGGCTACACCAACTGCAACGCGACCAATCCCAGAATTGCTGCTGATGCCGCCTATGCCTTTCATGTTCTTCCTCATTATCTCACTGCTATTTCTCCGAGCTCCCATGTTTGGCTTTGTCTCGCTACTCTGGTCTTTCATCACCTTACTGCTCTCTGATACTCTATATTTTTGCACGCTAGGATTTCTCTCTTTCTCACGCTCTTTCTTCATTGTAGCTCCATTTTTTGCTGAGGCCTCATGACGTCGACTCCTACTTTGTGCTATTGGAGCTGAAGAGATTGTGTCAGTTTCAGAGTGTTCAGTGGCAGAGCACTTAGATGCCCCTGAGGATTCAGGTTTGTCAACTGTGTTTAAGGATTGACTGCCAGAGAGGCCTCTATCTTTTCCTGCCTTGGAAAGTGATGGTGACCTTGCAGTACCCTTAGTTCCCGGACTTGTTGACTTAGGCCATGTCTTAGATGGTTTGGTGTTGCACGATACAGTCTTGTTTCCATCAGACGCTGGAGTCGCAGCTCCATGATAGAAATAAGGCATTGGTGTTGCTTTGAAATTAAGACTCTTCCTGAATTGTTTAATTTCAGCCTCAGTCTTTTCCTGAGTTTTTGCTTGGATCTGACTCATTTCTGCCGCTTTGGCATGCATTTTTTCCTCCAACTTCATGAAGAACTCTTTCCTCCTTTCTGCACGTTCATCACTTCTGAAACTGAAAGTTGCAGCACTCGGCTTCACGACTGGCTTAGTTGAATCAACAGTTTGTTTAGGCCTATTTGTGGTTTGACGCACTCTAGAATCAATCTTCTTTGTTGCAGTAGGTTGGGATTCAAGCATTTTCTTGGCCCTTGGCTCTTTTTCACTTTTATTCTGAACCTTCAATTTTGCAGCTGAACTTTCACAATCCTCTCTTTTTGGAGATCTCTGAATTGTACGTCTAACTGGAGTGGCTGTTCGCATTGATTGCTTCTCTGCTTTCAATCTTACCGGGCTCTCTTTTTCCTTTCTGTTCTGATTCTTTGCTCCAGATTTTGATGCCTCACGAGAAATGTGTTTGTGGACAGGGCTGACATTGACTGGAGAATTCATTCTAGGCTTTGTAGATTTATTTTCGACTGGAGCCCTCACCTTTGGAGAAAGACTTTGCCGGCGTTCTGAACTAGAATTACTAACAACTTTGCTGGTTGTTCCATTATTAAAGCACGATTCAGTTGGTTTGGATAATTCATCCACATCAACTGCATGATTGTTGAGGCACTTTTCTAATTCAATCTCTACTTTATCATTCCTCAAAAGCGACCTGTCACATCCAGTTTCTGTTTGATGTGCTTCCTCATGGTTAACATCTTCATGGACACTGTCCACCAGAATATCAGCATTTTCCAAAGCAGCTACCATCTGAGATTCATCAGGTGACACTGAAGAGTCCTCTCTTTCACATTCTATAACTCCAGCCTCTCCATGATACTCACAACTTTTGGGGCATTCATCGAAGTGAGCAAATTGGCCAGTTTCATTTACATAATCAGACTGGCTTCCTTCATTTACATTATCAAATTCCTGCACGTAATTTGCATTTTCGAAGACAATCTCGTGAAATTGATTATCAGGGCTGCTTTCATTTCCATTATTAAATTCTTCTCTGTAGTCTATGTTCTCCAGTCCATTCTCATTAAATTGATCAGAATTGCTAGCTTCAATTACAATATCAAATTCATCTCTGGAATCCTCATCATTGTCTAAGACATCATTTTCACCAGTTTGGTATTCTCTCTCATTTCGGCACTCAGATGAACCCTGAAGCAAAAGAGCCTTCCTTCTGAAATGAGCTTCAAAATAAGCTTTCTTTTCAATGACTGAACCCGGTTTTGAGCATTTCTCAACCTCTTCAAGATACCTATTGTGTGAGAAAGACGATCTCCTTTCCCAACATAAGGGTTCATTTTCAAACCTTCCAAATGAGATAGAACCGGAATGCAAAGAATCTATCTGAAAGTTGATGCTTAAAGGATCATCAATCTCTCCGGCCATTGTTTAACTCTCCCAAATCAAATGAAGCAGCAACCAACCATTATCTTTTATCACCCAGAAAAGATTAGATCTCAAACTCTTCAAAGAAGTAAACTTTTTTTTTTTTTTTTTTGTGAAATGTATAAAATCTAAAACATCCAAAAAACGAGGCAGCAGCTTTAACAAGTGAAAGTGTTTTTATATTTTTATTCTTGTTACGCAGATGAATGTATTGCAATGTGCGAGTTATCAATCAGTTCGAGTTGTGTCATGTGGTGGTGGGCGTCTTTTTGTCCTCTTCTGTCCGGACGCTACACAGTGAGGTATAATCTCTAGATTGACGGCTGGGATTTAAGATCGGAGGAGCAGAGCAGAGGTGTCAGCCAGTAGCGTCTACTGTTGACTTCCAGCTAATGATGTAAGAGAAACAGAAGTGTAATGTTAAGTCACAAGTGATTGTTATTTGTTTCTGGCAATTTTATCATGTAAC

*LcBI-1*

>Unigene0020640

GATCAATCAGGACACGAACAATAAAATATAGCTCTTTAACGAAAGGGCAGTATTGTAACTTGACACGGCTAGATATTTCGAACGAACCTCCTCAACGAACCGCTATAAATTCCATCTGTCTTCTCCATCAATAAATAAAAAGTTAATTCTTCGTCAATTTATAAAAAAGTCATTTAGCCGACGAATTTTCTCGCGGTTTCTCTGTACCCAAGCAACGATGGACGCGGCGTTCTCTTCCTTCTTCGATTCTCGACATGGATCGCGCTGGGACTCTTTCAAGAACTTTAACCAGATCTCACCCGTCGTGCAGTCCCACCTTAAACAGGTATATCTATCCTTATGTTGTGCACTGGTGGCATCAGCTACTGGAGTTTATCTGCATCTCCTGTGGAATATTGGTGGTATCCTTACCACATTTGCAATGATCGGATGCATTGCTTGGATGCTTTCAACTCCACTTTTCGAAGAGCGCAAAAGGGTTGGTCTATTGATGGCGGCTGCCCTTTTTGAAGGGGCTTCGATTGGTCCTCTCATTGAGTTGGCGATTCAAATTGACCCAAGTGTCCTGATCACGGCGTTCGTGGGAAGTGGGTTGGCCTTTGCATGTTTCTCAGTAGCAGCTATGGTAGCAAGGCGCAGAGAATATCTGTACCTCAGTGGCTTGCTTTCGTCTGGCTTGTCCATGCTTCTCTGGTTGCACTTTGCTTCCTCTATCTTTGGGGGTTCTACAGCGCTCTTCAAGTTTGAGTTGTACTTTGGGTTGTTGGTGTTCGTTGGCTACATTGTTGTGGACACTCAGGATATAATTGAGAAAGCTCACTTTGGTGACCTAGACTACGTGAAGCATGCCCTGACCCTTTTCACGGACTTTGTTGCTGTCTTTGTTCGCATTCTCATCATCATGCTGAAGAATGCAAATGAGAAGAATGAGAAGAAGAAGAAAAGGAGAAACTGAATATCTTAGTGGGTGGGTGGAACACTGCTACCTTTTGTTAACTATGGGTTGCAAAAAGCAAAGTTGTTGTCTATACAGTTAGTGTTTTGTTTTTCTTTTCCTTATTTCGAAGCTAAACCAGTGTTAACACTTCCAGATGTTTCATGTGTCTTGCTTAGTGGTGTTGGTAATTGTCTAATTTTGCTGATTGTAAAGGGTTTGAATCCTCCTGAAAACTGAAGTGCTGATTTCAGTTTTTTTCTCTAAATGAATTTACTGTTTGCAGTGTATGGCGTTGTACTAGTTTGAATTGATCTTATGGGTAATGGCAATTCTAGCCTTTTAGGTGGCCCTGCAAGTGTGATCCGGTGGAAGGAGTTCATCGAAGAAATTCATTATGTGTTTAGTGGAAAAACGCTGCGGGCCCCGAAAATTCGATGGGATTTTAAGTACTGTTTGTTTAGATAAACTTGTGAGGCACGAGTCGCACGACTCACGCGGAGAGAGAGGAAAAAACATGCTTGGAGCGGGGGTTTAGTATTTTATTGAGGGGCTGGATATTACGGG

*LcDAD-1*

>Unigene0017482

CAAAAGCTATGCCAAACAGGGCCTTCCAAAAGCTAGAGAGTATTTCTAGGTCTACGAGTTGGTCTGTGTAGAATTGATCTGTGCCAGAGAAAGCAGAGGATTATCCCAAATGGCGAGATCAACCGGTAAAGACGCTCAAGCTCTTTTCCACTCGCTTCGCTCTGCTTACGCTGCCACTCCTACCACTCTCAAGATCATCGATCTCTATGTGGGTTTCGCTGTCTTTACTGCTCTTATTCAGGTAGTGTACATGGCTACTGTTGGATCCTTCCCATTCAACTCTTTTCTTTCAGGGGTACTTTCTTGTGTTGGGACAGCAGTTCTAGCTGTTTGTCTCCGTATTCAAGTGAACAAGGAAAATAAGGAATTTAAGGATTTACCACCAGAGCGTGCTTTTGCGGACTTTGTTCTATGCAACTTGGTGCTGCATTTGGTCATTATGAATTTCCTTGGATAAGTTCAACTGTTTTATCTGCTGGAATGTTATTTCTATGGTTTATTAAGTGGGAGGAGAGTTGTGAAAGTAATTTACACCCGTTATGTGGGGAATTTTTCTTAGGTTATGACATACTATAAATGAAATGTAGAACACGGTAGTTGAGGCTTCTTCTGATTTACTTAAAAATTATTTACATAATCTTTTCCTTGCTGCATGAATCAGATTATTTTCTGGAGAAAATTAGTTGGTAACAAACATAAGAAAAGTCCATGTAATATTTTTCAATTACAT
